# Associations Among Obesity, Dietary Habits, and Erectile Dysfunction in Cardiac Patients: A Cross-Sectional Study

**DOI:** 10.3390/jcm15082946

**Published:** 2026-04-13

**Authors:** Małgorzata Biernikiewicz, Monika Stołyhwo-Gofron, Alina Kuryłowicz, Małgorzata Sobieszczańska, Krystyna Rożek-Piechura, Paulina Okrzymowska, Monika Markiewicz, Jana Gebala, Marzena Majchrowska, Dariusz Kałka

**Affiliations:** 1 Men’s Health Centre in Wrocław, 53-151 Wroclaw, Poland; malgorzata@biernikiewicz.pl (M.B.); markiewicz.moma@gmail.com (M.M.); janagebala@aol.com (J.G.); 2 Mediart Obesity and Overweight Treatment Clinic, Złota Karczma 25, 80-298 Gdansk, Poland; stolyhwo-gofron@wp.pl; 3 Department of Internal Medicine and Geriatric Cardiology, Centre of Postgraduate Medical Education, Orlowski Hospital, 00-416 Warsaw, Poland; alina.kurylowicz@cmkp.edu.pl; 4 Clinical Department of Geriatrics, Wroclaw Medical University, 50-369 Wroclaw, Poland; malsobie100@gmail.com; 5 Faculty of Physiotherapy, Wroclaw University of Health and Sport Sciences, 51-612 Wroclaw, Poland; krystyna.rozek-piechura@awf.wroc.pl (K.R.-P.); paulina.okrzymowska@awf.wroc.pl (P.O.); 6 Faculty of Medicine, Wroclaw University of Science and Technology, 50-370 Wroclaw, Poland; marzen89@gmail.com

**Keywords:** erectile dysfunction, obesity, adiposity, sexual dysfunction, diet, dietary intervention

## Abstract

**Background**: Obesity is a well-established risk factor for erectile dysfunction (ED), however, the association between specific dietary habits and sexual function among men with cardiac diseases remains insufficiently characterized. The objective of the present study was to analyze associations among obesity-related factors, dietary habits, and ED in cardiac patients. **Methods**: A cross-sectional analysis was performed in adult men with coronary artery disease (CAD). Erectile function was assessed using the International Index of Erectile Function (IIEF). Dietary habits were self-reported. Multivariable analyses were conducted to assess associations between demographic and dietary factors with IIEF scores. **Results**: 589 patients were included (mean age 60.1 ± 9.7 years; range 28–85). ED was present in 76% of participants, and 81% had excess body weight. Age demonstrated the strongest negative association with IIEF score. Adherence to a low-fat diet was associated with nearly a 2-point higher IIEF score, while each additional daily serving of vegetables was associated with an approximately 0.7-point increase in the IIEF score. **Conclusions**: Simple dietary modifications, particularly reduced fat intake and increased vegetable consumption, are associated with better erectile function in men with CAD. These findings suggest that dietary factors may be associated with erectile function; however, further prospective and interventional studies are needed to assess their clinical relevance.

## 1. Introduction

Obesity is a complex condition associated with numerous comorbidities. One of the obesity-related complications is erectile dysfunction (ED) [1,2]. A reduced testosterone level (hypogonadism) plays a key role in the link between obesity and obesity-related complications, such as ED. Several mechanisms contribute to reduced testosterone concentrations in obesity. One key mechanism is the hypogonadal-obesity cycle, which involves multiple interrelated hormonal feedback loops [3]. Adipocytes are characterized by a high aromatase activity, which enzymatically converts testosterone into estradiol, thereby reducing circulating androgen concentrations. Elevated estrogen levels, in turn, exert negative feedback on the hypothalamic–pituitary axis, suppressing gonadotropin-releasing hormone (GnRH) and luteinizing hormone (LH) secretion, ultimately leading to reduced gonadal testosterone production [4].

Dysregulation of leptin signaling represents an additional mechanism contributing to obesity-associated hypogonadism and ED. Leptin is a hormone produced by adipocytes and enterocytes of the small intestine that plays a key role in regulating body weight, food intake, and energy balance. Under normal physiological conditions, leptin also stimulates GnRH neurons in the hypothalamus, leading to the release of follicle-stimulating hormone (FSH) and LH, and subsequent stimulation of testicular testosterone production. LH acts directly on Leydig cells, which are responsible for testosterone synthesis [5]. In obesity, a state of leptin resistance develops. Despite elevated circulating leptin levels, target tissues, particularly in the brain, do not respond adequately to leptin signaling. As a result, the interaction between kisspeptin and its receptor in GnRH-producing neurons is impaired, preventing proper activation of GnRH secretion [6]. Consequently, instead of an increase in testosterone levels, a reduction is observed. In addition, high leptin concentrations further suppress testosterone production by acting directly on Leydig cells in the testes [7].

Multiple studies have demonstrated an inverse relationship between body weight, body mass index (BMI), or waist circumference and circulating testosterone levels, as well as a strong association between obesity and reduced sex hormone–binding globulin (SHBG) concentrations [8,9]. SHBG is a plasma protein synthesized in the liver that transports sex hormones, including testosterone and estrogen, in the circulation. Evidence indicates that obesity is associated with a marked reduction in SHBG levels, which in turn contributes to lower circulating testosterone concentrations. Notably, the association between BMI and reduced SHBG is stronger than that between aging and increased SHBG; BMI explains approximately threefold more variability in SHBG levels than age alone. In a large cohort of 1547 infertile men, each one-unit increase in BMI was associated with a decrease in SHBG of 1.1 nmol/L [10]. Similarly, in weight-stable men, SHBG levels showed a negative linear relationship with BMI, declining by approximately 0.2 nmol/L per unit increase in BMI [11]. SHBG also decreases in response to obesity-related factors, including reduced insulin sensitivity and increased inflammatory mediators such as tumor necrosis factor α (TNF-α), myeloperoxidase, and interleukin 6 (IL-6) [12,13]. Consequently, obesity-related reductions in SHBG are considered one of the key mechanisms underlying decreased testosterone levels.

Excessive accumulation of adipose tissue, as seen in obesity, initiates a chronic low-grade systemic inflammatory state that actively reinforces further weight gain, creating a self-perpetuating vicious cycle [5]. In this context, adipose tissue functions as an immunologically active organ, releasing pro-inflammatory signals that promote insulin resistance and metabolic dysregulation [14]. Inflammatory and metabolic pathways are closely intertwined, as evidenced by findings showing that each standard deviation increase in insulin levels is associated with a 0.26 standard deviation increase in body mass, underscoring the strength of this feedback loop. Progressive adiposity is positively associated with systemic inflammation and inversely associated with testosterone concentrations, with higher C-reactive protein levels correlating with lower testosterone levels [15,16]. Reduced testosterone further exacerbates fat accumulation and inflammation, thereby sustaining the cycle. This inflammatory milieu promotes endothelial dysfunction, oxidative stress, prothrombotic changes, and accelerated atherosclerosis, explaining why higher BMI is associated with more subclinical and clinical cardiovascular disease even after adjusting for traditional risk factors. In addition, low serum testosterone is independently associated with higher rates of metabolic syndrome, type 2 diabetes, dyslipidemia, and systemic inflammation, which are major drivers of cardiovascular disease [17]. Importantly, testosterone replacement therapy has been shown to reduce inflammatory markers, including C-reactive protein, interleukin-1β, and tumor necrosis factor-α, indicating a potential strategy for interrupting this pathological feedback loop [18].

**Figure 1:**
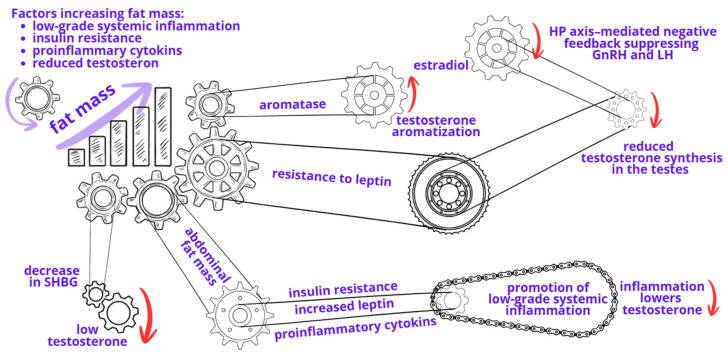
The hypogonadal-obesity cycle: interactions between adiposity and testosterone deficiency (created with Canva v. 1.121.0). Red arrows indicate mechanisms contributing to reduced testosterone levels, whereas purple arrows indicate links between risk factors and increased fat mass.

Figure 1 schematically illustrates the interrelated pathophysiological mechanisms linking excess adipose tissue to reduced testosterone levels, highlighting the self-perpetuating vicious cycle among adiposity, inflammation, hormonal dysregulation, and metabolic impairment.

On the other hand, accumulating evidence highlights the role of diet in promoting weight reduction and improving sexual function. The strongest evidence supports the Mediterranean diet [19]; however, beneficial effects have also been reported for general dietary modifications [20], or low-fat, high-protein or high-carbohydrate dietary patterns [21,22]. Obesity is a chronic disease characterized by periods of remission and relapse [23]. Its multifactorial etiopathogenesis, the limited long-term effectiveness of current treatments, and the challenges patients face in maintaining sustainable lifestyle changes underscore the need for additional therapeutic strategies.

Dietary interventions are relatively easy to implement and may be effective, particularly when patients are aware of and understand their benefits. In this retrospective study, we aimed to examine the habitual dietary patterns of men with coronary artery disease (CAD). Accordingly, the objective of the present study was to analyze the associations among obesity, dietary habits, and ED in patients with CAD. We further sought to identify simple, practical dietary patterns linked to body weight and sexual function.

## 2. Materials and Methods

### 2.1. Study Design

This retrospective, cross-sectional observational study included patients with CAD undergoing rehabilitative treatment at cardiac rehabilitation centers. Data were collected between April 2022 and November 2025. The study was conducted following approval from the Bioethics Committee. Written informed consent for the retrospective analysis of medical and questionnaire data for scientific purposes was included in the patient documentation and obtained from all patients. The study was conducted as part of the PREVANDRO project, an educational program focused on cardiosexology.

### 2.2. Sources of Data and Questionnaires

Medical records served as the source of anthropometric data (particularly parameters related to obesity risk), demographic characteristics, modifiable risk factors, and comorbid conditions. Patients were stratified into groups according to clinically relevant body mass index (BMI) thresholds [24]. The presence of ED was assessed using the abridged International Index of Erectile Function (IIEF-5) questionnaire, which consists of five items scored from 1 to 5, yielding a total score ranging from 5 to 25. ED was defined as a total score of ≤21, based on sexual activity during the preceding four weeks in the absence of pharmacological support for sexual performance [25]. Diet was evaluated based on a dedicated questionnaire on dietary habits. A low-fat diet was defined as a reduction in total fat intake, with particular emphasis on limiting saturated fatty acids. A low-salt (sodium-restricted) diet was defined as reduced sodium intake, primarily achieved by limiting processed foods and avoiding added salt. A low-sugar diet was defined as reduced intake of free and added sugars, especially from sugar-sweetened beverages and ultra-processed products. A portion of vegetables was defined as approximately 80 g, in line with international dietary recommendations from the European Commission [26].

Participants completed the questionnaire themselves; however, an interviewer was available to clarify any questions if needed. Respondents were assured of full confidentiality and completed the questionnaire without influence from third parties. The questionnaire comprised only closed-ended questions. All collected data were anonymized and coded, ensuring full protection of participants’ personal information.

### 2.3. Patients and Eligibility Criteria

Inclusion criteria comprised adult patients (≥18 years) with CAD undergoing cardiac rehabilitation. In all patients, CAD was confirmed by angiographic evidence of coronary stenosis. Exclusion criteria included ED secondary to surgical treatment. Patients were also excluded if they had a history of abdominal aortic or iliac artery repair, neurological treatment following a central nervous system vascular event, or orthopedic treatment for spinal or pelvic injuries. Additional exclusion criteria were current psychiatric care, antidepressant therapy, pulmonary treatment for diseases impairing respiratory function, and hormonal therapy with GnRH agonists or androgens.

### 2.4. Statistical Analysis

Data were statistically analyzed using MedCalc v. 23.2.08 (MedCalc Software Ltd., Ostend, Belgium). Summary statistics included means with standard deviations (SD), as well as counts and percentages. The distribution of continuous variables was assessed using the Shapiro–Wilk test. Differences in continuous variables between BMI-stratified groups were analyzed using one-way analysis of variance (ANOVA) for normally distributed data or the Kruskal-Wallis test for non-normally distributed variables, while categorical variables were compared using the chi-square test. Least-squares multiple regression analysis was performed to assess associations between dietary habits and outcome variables. Missingness was below 2% for all variables except for the number of meals (5.6%) and alcohol consumption (10.0%). Given the low level of missing data and the large sample size, analyses were performed using complete case analysis only. Differences were considered statistically significant at a value of *p* < 0.05.

## 3. Results

### 3.1. Patients

A total of 589 patients with CAD were included in the study. Participants ranged in age from 28 to 85 years, with a mean age of 60.11 ± 9.68 years. The IIEF-5 scores ranged from 5 to 25, with a mean value of 15.72 ± 6.40. BMI values ranged from 19.81 to 42.92 kg/m^2^, with a mean of 28.32 ± 3.70 kg/m^2^. Smoking was reported by 76.8% of participants. The characteristics of the study group, including comorbidities and current treatment are presented in Table 1.

ED was present in 76% of patients: 24.2% had mild ED, 25.0% had moderate ED, 10.7% had moderate-to-severe ED, and 16.0% had severe ED. In contrast, 24% of the study population did not report any symptoms of ED. Obesity was observed in 31.9% of patients, 49.1% were overweight, and 19.0% had normal body weight. The BMI-stratified groups did not differ significantly with respect to age, IIEF score, the prevalence of ED, or smoking status (Table 2).

### 3.2. Dietary Patterns

Dietary habits reported by the study participants indicated partial adherence to recommended nutritional practices. A low-fat diet was declared by 73.2% of patients, while 71.2% reported limiting salt intake, and 49.1% reported reducing sugar consumption. Regular breakfast consumption was common, with 87.9% of participants reporting regular breakfast consumption. Snacking between meals was reported by 56.5% of patients, and 67.6% declared regular meal patterns. The mean number of meals consumed per day was 3.52 ± 0.86, ranging from 1 to 7 meals. Participants reported a mean daily intake of 3.74 ± 0.84 servings of vegetables, with values ranging from 1 to 5 servings. Alcohol consumption was widespread, as only 0.4% of participants reported complete abstinence; the mean frequency of alcohol intake was 3.63 ± 0.74 times per week, with a range from 0 to 4.

With respect to differences across BMI categories, men with normal body weight reported more regular meal patterns, less frequent snacking, higher vegetable consumption, and lower alcohol intake. However, statistically significant differences between BMI groups were observed only for meal regularity and sugar consumption (Table 3).

Next, patients were stratified into four groups according to BMI category and the presence of ED. Normal body weight was defined as BMI < 25 kg/m^2^, while increased BMI was defined as BMI ≥ 25 kg/m^2^. Absence of ED was defined by an IIEF-5 score of 22 and above, whereas the presence of ED was defined by an IIEF-5 score of ≤21. This comparison indicates that men with normal body weight ate more regularly, regardless of the presence of ED. A significant difference was also observed in vegetable consumption: men with normal body weight and no ED consumed significantly more vegetables than men with elevated body weight and ED (Table 4).

### 3.3. Regression Analysis

We investigated factors influencing the IIEF score using a regression model. After adjustment for all analyzed variables, each additional year of age was associated with a 0.27-point decrease in the IIEF score. Conversely, holding all other variables constant, each additional daily serving of vegetables was associated with a 0.67-point increase in the IIEF score (Table 5). Diagnostic parameters confirmed that the overall model was statistically significant (F = 10.28, *p* < 0.0001), with an R^2^ of 0.2065 and an adjusted R^2^ of 0.1864. The residual standard deviation was 5.74. Residual normality, assessed with the Shapiro–Wilk test, indicated deviation from normality (W = 0.9707, *p* < 0.0001).

To further explore associations potentially attenuated by the strong effect of age, we additionally constructed a complementary model excluding age from the analysis. When age was excluded from the model and only dietary habits were considered, several factors showed a significant association with the IIEF score. After adjustment for the remaining variables, patients reporting adherence to a low-fat diet had IIEF scores nearly 2 points higher than those who did not follow such a diet. In contrast, patients who reported eating breakfast had IIEF scores that were 1.9 points lower. Additionally, each additional daily serving of vegetables was associated with an increase of approximately 0.7 points in the IIEF score (Table 6). Diagnostic parameters confirmed that the overall model was statistically significant, although its explanatory power was modest. The regression model reached significance (F = 3.58, *p* = 0.0005), with an R^2^ of 0.051 and an adjusted R^2^ of 0.037. The residual standard deviation was 6.25. Assessment of residual normality with the Shapiro–Wilk test showed deviation from normality (W = 0.9579, *p* < 0.0001).

## 4. Discussion

In this cohort of men with CAD, with a mean age of approximately 60 years, ED was present in 76% of patients, and elevated body weight was observed in 81%. Age emerged as the strongest determinant of erectile function, with each additional year associated with a 0.27-point decrease in the IIEF score. After adjustment for dietary covariates, dietary factors also showed significant associations with erectile function. Patients reporting adherence to a low-fat diet had IIEF scores nearly 2 points higher than those not following such a diet. In contrast, breakfast consumption was associated with a 1.9-point lower IIEF score. Furthermore, each additional daily serving of vegetables was associated with an approximately 0.7-point increase in the IIEF score. Although these effect sizes were modest in relation to the minimal clinically important difference (considered approximately 4 for the erectile domain on the IIEF scale) [27], they may still be relevant at the population level and support the potential importance of cumulative dietary behaviors in this high-risk group.

Despite established links between obesity and ED, and emerging evidence on the role of overall dietary patterns, data on the association between specific dietary habits and erectile function remain limited, particularly in cardiac patients. Previous studies have largely focused on weight reduction or predefined dietary models, rather than individual dietary behaviors assessed in real-world settings. In this context, the present study provides novel insight by evaluating multiple dietary habits in relation to erectile function and obesity in a cohort of men undergoing cardiac rehabilitation, thereby addressing an important gap in the literature and offering clinically applicable findings.

Diet represents a simple yet relatively understudied intervention in both cardiovascular disease and ED, and patient adherence to dietary recommendations remains low. This is unfortunate, as dietary modification is among the least costly interventions and confers broad benefits for overall health. Dardzińska et al. [28] evaluated adherence to the European Society of Cardiology (ESC) dietary guidelines in a cohort of 1244 current or former smokers at increased cardiovascular risk. Only 2% of participants reported consuming more than two daily servings of both fruits and vegetables, and merely 3% reported daily nut consumption. The majority of individuals were overweight, consumed insufficient dietary fiber, and derived excessive energy from total and saturated fats. The mean animal-to-plant protein ratio exceeded recommended levels, as did the omega-6 to omega-3 fatty acid ratio. Among participants with arterial hypertension, diabetes, or CAD, only 40% reported a daily cholesterol intake below 200 mg, and just 12% consumed less than 7% of total energy from saturated fats. Al Daccache et al. [29] investigated adherence to dietary, pharmacological, and physical activity recommendations among 367 Lebanese patients with cardiovascular disease. Poor adherence was observed in approximately 43% of patients for dietary recommendations, 70% for medication use, and 52% for physical activity. Lower mental well-being emerged as a significant predictor of poor adherence to both dietary and pharmacological recommendations. Interestingly, overweight and obesity were associated with higher odds of dietary adherence. Efremova and Shutov [30] assessed adherence to dietary recommendations among 214 elderly patients with cardiovascular comorbidity and chronic kidney disease (CKD), with a mean age of 69.5 ± 7.6 years. Daily consumption of vegetables and fruits was reported by only 56.3% of patients, while 52% included fish in their diet, either daily or at least twice a week. Adequate intake of vegetables and fruits (4–6 servings per day) was observed in only 24% of participants. Although 65.4% of elderly patients reported having scales at home for self-monitoring of body weight, over 50% stated that they never monitored their weight. Collectively, these findings highlight alarmingly low adherence to dietary recommendations, underscoring a substantial unmet need for improved patient education and the implementation of structured, effective dietary interventions.

In the present study, late breakfast consumption or skipping breakfast showed an unexpected negative association with IIEF scores (regular breakfast consumption was associated with a 1.9-point lower IIEF score). Given that erectile dysfunction shares many risk factors with cardiovascular disease, regular breakfast consumption might be expected to exert beneficial effects. However, evidence regarding breakfast habits and cardiometabolic outcomes is inconsistent. A meta-analysis of randomized controlled trials by Sievert et al. [31], including seven studies, found a small difference in body weight favoring participants who skipped breakfast (mean difference 0.44 kg; 95% CI 0.07–0.82), although those assigned to breakfast consumption had higher total daily energy intake. In contrast, a meta-analysis by Zhang et al. [32] reported that skipping breakfast was associated with a higher risk of cardiovascular disease (OR 1.17; 95% CI 1.09–1.26), including CAD (OR 1.14; 95% CI 1.05–1.24), stroke (RR 1.15; 95% CI 1.01–1.30), and cardiovascular mortality (OR 1.49; 95% CI 1.20–1.84). Importantly, breakfast timing should not be evaluated in isolation. Lifestyle factors such as sleep patterns and circadian behaviors may influence breakfast habits; for example, Sato et al. [33] demonstrated significant associations between wake-up time, bedtime, and the frequency of breakfast skipping. Similarly, Palomar-Cros et al. [34] reported that the combination of longer nighttime fasting and earlier breakfast was associated with a lower risk of prostate cancer compared with shorter fasting and later breakfast. In our study, additional lifestyle factors related to sleep and circadian patterns were not assessed, which may have influenced the observed associations and highlighted the need for further research in this area.

Experts unanimously recommend monitoring testosterone levels and promoting weight reduction in patients with obesity. The American Association of Clinical Endocrinologists and the American College of Endocrinology Comprehensive Clinical Practice Guidelines for the Medical Care of Patients with Obesity [35] advise that all men with increased waist circumference (>94 cm) or obesity should be evaluated for hypogonadism based on medical history, physical examination, and measurement of serum testosterone levels. Conversely, all men diagnosed with hypogonadism should be assessed for the presence of overweight or obesity. According to these recommendations, management of hypogonadism in men with increased waist circumference or obesity should primarily include interventions aimed at weight reduction, such as increased physical activity and dietary modification. Clinically meaningful improvements in serum testosterone concentrations have been observed with weight loss exceeding 5–10%. In this population, testosterone supplementation has been shown to promote weight reduction, decrease waist circumference, and improve metabolic parameters, including fasting glucose, glycated hemoglobin (HbA1c), lipid profile, and blood pressure. The European Society of Endocrinology and the European Academy of Andrology [36,37] emphasize the central role of obesity in the development of hypogonadism. In cases of functional hypogonadism associated with obesity, they recommend considering testosterone replacement therapy in men with hypogonadism and coexisting sexual dysfunction to improve libido, erectile function, and sexual satisfaction.

The present study indicates that simple dietary modifications, namely reducing fat intake and increasing vegetable consumption, are associated with better sexual function, whereas regular meal patterns and breakfast consumption did not confer additional benefit. Liu et al. [38] reviewed the effects of major dietary patterns on testosterone levels and their underlying mechanisms, focusing on ketogenic, vegetarian, Mediterranean, and Western diets. They demonstrated beneficial effects of ketogenic, vegetarian, and Mediterranean dietary patterns, while reporting adverse effects associated with Western diets. Despite the well-established association between obesity and ED and the alarming global increase in obesity prevalence, awareness of this relationship remains limited in both clinical practice and patient education. This gap is particularly concerning given the higher prevalence of ED observed in older individuals and in those with additional cardiometabolic risk factors [8,39].

Evidence regarding the effect of ketogenic diets on testosterone levels remains limited and inconsistent. A meta-analysis by Furini et al. [40] reported a significant increase in total testosterone following ketogenic dietary interventions; however, this analysis included only 111 participants, and the observed testosterone increase was significantly correlated with the magnitude of weight loss. Additionally, a larger meta-analysis by Ji et al. [41], encompassing 44 randomized controlled trials, demonstrated that ketogenic diets significantly reduced systemic inflammation, with decreases in tumor necrosis factor-α (TNF-α) of 0.32 pg/mL and interleukin-6 (IL-6) of 0.27 pg/mL. Notably, these anti-inflammatory effects were more pronounced in individuals with a body mass index > 30 kg/m^2^ than in those with a BMI ≤ 30 kg/m^2^. These mechanisms may contribute to improvements in testosterone levels; however, despite promising findings, long-term human studies are required to definitively clarify their role in testosterone metabolism.

A vegetarian or predominantly plant-based diet has been shown to significantly reduce the risk of cardiovascular disease by improving modifiable cardiovascular risk factors that contribute to organic erectile dysfunction, including obesity, hypertension, diabetes, dyslipidemia, and poor glycemic control. Evidence indicates that such dietary patterns are associated with reductions in body weight, blood pressure, glycated hemoglobin, and low-density lipoprotein cholesterol, thereby supporting both cardiovascular and sexual health [42]. Phytochemical compounds may improve endothelial function by reducing reactive oxygen species and increasing nitric oxide bioavailability, thereby promoting anti-inflammatory and antioxidative effects [43]. However, vegetarian diets have also been linked to a higher risk of frailty compared with omnivorous diets, largely due to potential inadequacies in protein, vitamin D, and vitamin B12 intake, which may result in reduced lean body mass [44]. Adequate protein intake is particularly important for preserving skeletal muscle and pelvic floor muscle function, which play a key role in achieving and maintaining a rigid erection [45]. In the present study, higher daily vegetable consumption was independently associated with higher IIEF score. These findings are consistent with those of Wang et al. [46], who reported a 10% reduction in erectile dysfunction risk with each additional daily serving of fruits or vegetables, after adjustment for confounders. Similarly, Ramirez et al. [47] demonstrated an inverse association between daily vegetable consumption and erectile dysfunction (OR 0.47; 95% CI 0.28–0.77). The broader cardiovascular benefits of fruit and vegetable intake were confirmed in a large meta-analysis by Dauchet et al. [48], including nearly 200,000 participants, which showed a 4% reduction in coronary heart disease risk per additional daily portion of fruits and vegetables and a 7% reduction per additional portion of fruit alone. These data reinforce the role of increased plant food consumption in reducing modifiable cardiovascular risk factors and supporting erectile function, while highlighting the importance of ensuring adequate protein and micronutrient intake within vegetarian dietary patterns.

The traditional Mediterranean diet, originally described in populations from Greece and Southern Italy in the early 1960s, represents a comprehensive dietary pattern that extends beyond high vegetable intake alone [49]. It is characterized by high consumption of fruits (3–4 servings/day) and vegetables (2–3 servings/day), a high monounsaturated-to-saturated fat ratio (≥2), daily intake of whole grains and legumes, moderate consumption of fish and nuts (3–5 servings/week), moderate wine intake (1–2 glasses/day), low intake of red and processed meat (4–5 servings/month), and daily consumption of low-fat dairy products. Compared with strictly vegetarian diets, this pattern provides a broader spectrum of bioactive nutrients, including omega-3 fatty acids, high-quality protein, and polyphenols. Giugliano et al. [50] demonstrated a significant, graded association between adherence to the Mediterranean diet and sexual activity in men, with the proportion of sexually active individuals increasing across tertiles of adherence (from 65.1% to 74.4%, *p* = 0.01). Men with the highest adherence also had a lower prevalence of overall erectile dysfunction (51.9% vs. 62%, *p* = 0.01) and severe erectile dysfunction (16.5% vs. 26.4%, *p* = 0.01) compared with those with low adherence. Consistent with these findings, the present study observed that adherence to a low-fat dietary pattern was associated with better sexual function. The beneficial effects of the Mediterranean and low-fat dietary patterns on erectile function are likely mediated through improvements in endothelial function, reductions in systemic inflammation, and favorable metabolic profiles, reflecting the advantages of a nutritionally diverse dietary approach over vegetable-based patterns alone [51].

Alcohol consumption showed a negative but non-significant association with ED in the present study, which may reflect the high prevalence of alcohol use in the study population and limited contrast between exposure groups. Prior studies suggest a more nuanced relationship. Li et al. [52] reported a significant association between regular alcohol consumption and ED (OR 0.89; 95% CI 0.81–0.97), describing a J-shaped pattern. Similarly, a meta-analysis of population-based studies by Cheng et al. [53] found that regular alcohol consumption was inversely associated with ED (OR 0.79; 99% CI 0.67–0.92; *p* < 0.001). In that analysis, consumption of eight or more drinks per week was associated with a modestly reduced risk of ED (OR 0.85; 99% CI 0.73–0.99; *p* = 0.007), whereas lower intake levels (1–7 drinks per week) were not significantly associated with ED risk. Interpretation of these findings is complicated by substantial methodological limitations inherent to alcohol research, including reliance on self-reported intake, recall bias, underreporting, and cultural influences on disclosure [54,55]. Moreover, emerging large-scale and genetic epidemiological data challenge the notion of alcohol-related health benefits. An analysis of 371,463 participants from the UK Biobank by Biddinger et al. [56] demonstrated a nonlinear but consistently risk-increasing association between habitual alcohol consumption and cardiovascular disease, with risk rising even at low levels of intake. Genetic analyses further suggested that alcohol consumption at any level is associated with increased cardiovascular risk, although the magnitude of risk varies across consumption levels, including those considered acceptable by current guidelines. Taken together, these data indicate that any potential protective association between alcohol intake and ED should be interpreted with caution, particularly in light of the broader adverse cardiovascular effects of alcohol consumption.

**Figure 2:**
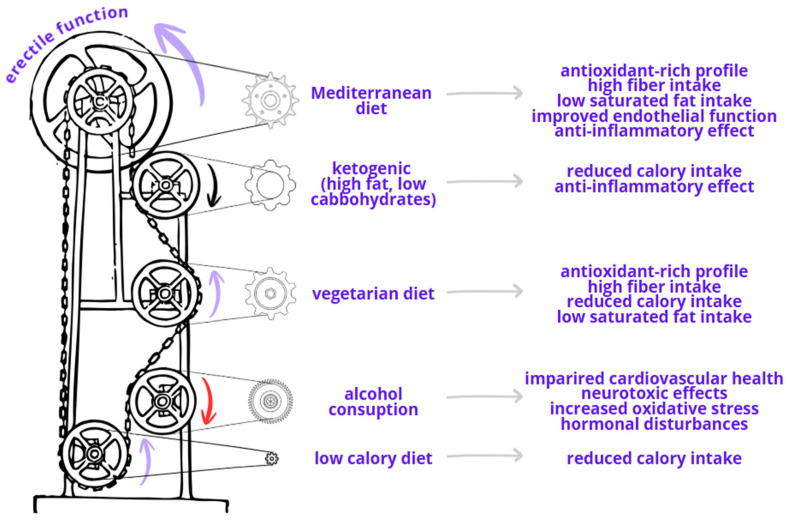
Schematic overview of diet-related mechanisms influencing erectile function (created with Canva v. 1.121.0). Purple arrow denotes positive impact, black arrow denotes limited evidence, and red arrow indicates negative impact.

A schematic overview of the potential biological mechanisms linking dietary patterns with erectile function is presented in Figure 2.

### 4.1. Clinical Relevance

The findings of this study have several clinically relevant implications for the management of men with coronary artery disease. First, the very high prevalence of ED (76%) observed in this cohort reinforces the need for routine assessment of sexual health in cardiovascular care, as ED may represent an important marker of overall vascular status. Importantly, while age remained the dominant determinant of erectile function, certain modifiable lifestyle factors, such as vegetable consumption, were associated with higher IIEF scores, even after adjustment for multiple variables. Although causality cannot be inferred due to the cross-sectional design, these associations suggest that simple dietary habits may serve as accessible targets for patients undergoing cardiac rehabilitation programs. These results support the integration of dietary assessment and counseling into comprehensive, multidisciplinary management of patients with CAD, with potential benefits extending beyond cardiovascular outcomes to include sexual health.

### 4.2. Limitations

Several limitations of this study should be acknowledged. The observational and cross-sectional design precludes causal inference between dietary habits and erectile function. Although associations were identified between specific dietary patterns and better sexual function, reverse causation cannot be excluded, as men with better overall health or sexual function may be more likely to adhere to healthier diets.

Dietary intake was assessed using self-reported data, which are inherently subject to recall bias, social desirability bias, and misclassification. This limitation is particularly relevant for variables such as alcohol consumption, fat intake, and meal patterns, which may be underreported or inaccurately quantified. This approach, while pragmatic, may not fully capture the complexity, quality, and synergistic effects of whole dietary patterns. The lack of detailed nutritional assessment, including portion size estimation, macronutrient distribution, and total caloric intake, limits the precision of dietary exposure characterization. Moreover, potential collinearity and interdependence among dietary variables may have influenced the stability of regression estimates; however, due to the use of simplified, self-reported dietary data, it was not feasible to apply advanced dietary pattern analyses.

Next, nutritional status was assessed using BMI, a widely used parameter that does not reflect body composition, which is a key factor in cardiometabolic risk assessment.

It should also be noted that testosterone levels were not directly measured in this cohort, which limited mechanistic insight into the potential hormonal pathways linking diet, obesity, and erectile function. Instead, erectile function was assessed using the self-reported IIEF-5 questionnaire. Because testosterone plays a central role in sexual health and is strongly influenced by body composition and dietary patterns, the absence of hormonal data limits the interpretation of biological plausibility.

Finally, the study population consisted of men with CAD and a high prevalence of overweight and obesity, which may limit the generalizability of the findings to younger, healthier populations or to men without established cardiometabolic disease. Similarly, the high prevalence of alcohol consumption in the cohort may have reduced the ability to detect meaningful associations between alcohol intake and ED. Smoking was highly prevalent in the study population, and although smoking status was included in the multivariable analysis, residual confounding cannot be excluded.

Despite these limitations, the study provides clinically relevant insights into simple, potentially modifiable dietary behaviors associated with erectile function in a high-risk population and underscores the need for well-designed prospective and interventional studies with detailed nutritional and hormonal assessments.

## 5. Conclusions

Obesity and ED are highly prevalent among men with CAD, underscoring the close interrelationship between cardiometabolic health and sexual function. In this study, simple dietary modifications, particularly reduced fat intake and higher vegetable consumption, were associated with better erectile function. The observed associations warrant further investigation in prospective and interventional studies before any clinical recommendations can be made.

## Figures and Tables

**Table 1:** Characteristics of the study group.

Variable	Total
Age, years; mean ± SD	60.11 ± 9.68
BMI, kg/m^2^; mean ± SD	28.32 ± 3.70
Erectile dysfunction	
Severe ED (5–7 scores on IIEF-5), *n* (%)	94 (16.01)
Moderate-to-severe ED (8–11 scores on IIEF-5), *n* (%)	63 (10.71)
Moderate ED (12–16 scores on IIEF-5), *n* (%)	147 (25.04)
Mild ED (17–21 scores on IIEF-5), *n* (%)	142 (24.19)
Without ED (22–25 scores on IIEF-5), *n* (%)	141 (24.02)
Pharmacotherapy	
Beta-blockers, *n* (%)	518 (87.9)
Angiotensin-converting-enzyme inhibitors, *n* (%)	403 (68.4)
Angiotensin II receptor blockers, *n* (%)	61 (10.4)
Statins, *n* (%)	523 (88.8)
Calcium channel blockers, *n* (%)	115 (19.5)
Diuretics, *n* (%)	201 (34.1)
Alfa-blockers, *n* (%)	21 (3.6)
Comorbidities	
Hypertension, *n* (%)	447 (75.9)
Diabetes, *n* (%)	111 (18.8)
Dyslipidemia, *n* (%)	151 (25.6)
Clinical data related to CAD	
Left ventricular diameter (LVD), mm, mean ± SD	54.42 ± 6.82
Left atrial diameter (LA), mm, mean ± SD	42.29 ± 5.27
Ejection fraction (EF), %, mean ± SD	54.52 ± 9.79
Right ventricular diameter (RVD), mm, mean ± SD	26.72 ± 5.22
Status post myocardial infarction, *n* (%)	373 (63.3)
Status post PCI, *n* (%)	336 (57.0)
Status post CABG, *n* (%)	231 (39.2)
Status post stroke, *n* (%)	9 (1.5)

CABG, coronary artery bypass graft; CAD, coronary artery disease; BMI, body mass index; ED, Erectile dysfunction; IIEF-5, International Index of Erectile Function 5; PCI, percutaneous coronary intervention.

**Table 2:** Comparison of age, erectile function, prevalence of erectile dysfunction, and smoking across BMI categories.

Variable	Normal (<25) *n* = 112	Overweight (25 to <30) *n* = 289	Obesity (≥30) *n* = 188	*p*-Value
Age, years	61.23 ± 9.88 (range: 40–83)	60.23 ± 9.78 (range: 28–85)	59.26 ± 9.36 (range: 32–80)	*p* = 0.4884
IIEF-5 score	15.78 ± 6.62 (range: 5–25)	15.71 ± 6.49 (range: 5–25)	15.70 ± 6.14 (range: 5–25)	*p* = 0.9456
Presence of ED	72.07%	75.35%	79.26%	*p* = 0.3504
Smoking	77.27%	73.96%	80.85%	*p* = 0.2176

ED, Erectile dysfunction; IIEF-5, International Index of Erectile Function 5.

**Table 3:** Comparison of dietary patterns across BMI categories.

Variable	Normal (<25)	Overweight (25 to <30)	Obesity (≥30)	*p*-Value
Low fat	75.5%	72.9%	72.3%	0.8321
Low salt	69.1%	68.7%	76.1%	0.1972
Low sugar	37.3%	50.0%	54.8%	**0.0130**
Breakfast	88.9%	87.8%	87.6%	0.9400
Regular meals	79.1%	65.9%	63.4%	**0.0143**
Number of meals	3.61 ± 0.81	3.50 ± 0.86	3.49 ± 0.88	0.3053
Snacking	50.5%	55.3%	61.8%	0.1373
Vegetable servings	3.90 ± 0.83	3.72 ± 0.81	3.71 ± 0.88	0.1237
Alcohol per week	3.53 ± 0.87	3.63 ± 0.74	3.67 ± 0.67	0.5645

BMI, body mass index. Bold denotes statistical significance.

**Table 4:** Comparison of dietary patterns across BMI and ED categories.

Variable	Normal Weight; No ED	Normal Weight; ED	Increased Weight; No ED	Increased Weight; ED	*p*-Value
Low fat	80.6%	73.4%	83.6%	69.4%	0.0215
Low salt	80.6%	64.6%	73.6%	71.0%	0.3382
Low sugar	45.2%	34.2%	45.5%	53.8%	**0.0113**
Breakfast	87.1%	89.5%	85.3%	88.4%	0.8138
Regular meals	71.0%	82.1%	58.7%	66.7%	**0.0087**
Number of meals	3.57 ± 0.69	3.55 ± 0.75	3.54 ± 0.84	3.47 ± 0.87	0.5165
Snacking	51.6%	49.4%	59.8%	57.2%	0.4805
Vegetable servings	4.07 ± 0.81 (a)	3.88 ± 0.87	3.85 ± 0.79	3.69 ± 0.85 (a)	0.0430 *
Alcohol per week	3.39 ± 1.07	3.58 ± 0.80	3.59 ± 0.80	3.66 ± 0.69	0.6321

BMI, body mass index; ED, erectile dysfunction. Normal weight was defined as BMI < 25 kg/m^2^, while increased weight as BMI ≥ 25 kg/m^2^. * Kruskal-Wallis test: (a) groups with the same letter differ significantly in the post-hoc test according to Conover. Bold denotes statistical significance.

**Table 5:** Multivariable regression analysis of factors associated with IIEF-5 score (*n* = 487).

Independent Variables	Coefficient	Std. Error	95% CI	t	*p*-Value
(Constant)	32.2843	3.3069	25.7862 to 38.7823	9.7626	**<0.0001**
Age	−0.2653	0.02873	−0.3218 to −0.2089	−9.2352	**<0.0001**
BMI	−0.04289	0.07213	−0.1846 to 0.09885	−0.5946	0.5524
Low-fat diet	1.0204	0.6845	−0.3246 to 2.3655	1.4907	0.1367
Low-salt diet	0.4969	0.6767	−0.8328 to 1.8266	0.7343	0.4632
Low-sugar diet	−0.5494	0.5805	−1.6900 to 0.5912	−0.9465	0.3444
Regular meals	−0.6199	0.6164	−1.8311 to 0.5912	−1.0058	0.3150
Snacking	−0.005965	0.5418	−1.0706 to 1.0587	−0.01101	0.9912
Breakfast	−0.2325	0.8625	−1.9274 to 1.4624	−0.2695	0.7877
Number of meals	−0.07565	0.3301	−0.7243 to 0.5730	−0.2292	0.8188
Vegetable consumption	0.6726	0.3161	0.05139 to 1.2938	2.1275	**0.0339**
Alcohol consumption	−0.3160	0.3571	−1.0177 to 0.3857	−0.8849	0.3766
Smoking	−0.8625	0.6430	−2.1260 to 0.4011	−1.3412	0.1805

BMI, body mass index; IIEF-5, International Index of Erectile Function 5. Bold denotes statistical significance.

**Table 6:** Multivariable regression analysis of dietary habits associated with IIEF-5 score (*n* = 589).

Independent Variables	Coefficient	Std. Error	95% CI	t	*p*
(Constant)	13.4562	1.7179	10.0815 to 16.8309	7.8330	<0.0001
Low-fat diet	1.9887	0.7033	0.6072 to 3.3702	2.8278	**0.0049**
Low-salt diet	0.3069	0.7035	−1.0752 to 1.6889	0.4362	0.6629
Low-sugar diet	−1.0316	0.5816	−2.1740 to 0.1108	−1.7739	0.0767
Breakfast	−1.9060	0.8955	−3.6653 to −0.1468	−2.1284	**0.0338**
Number of meals	0.1927	0.3313	−0.4581 to 0.8436	0.5817	0.5610
Snacking	0.4474	0.5531	−0.6390 to 1.5339	0.8090	0.4189
Regular meals	−1.1133	0.6377	−2.3660 to 0.1394	−1.7458	0.0814
Vegetable consumption	0.7092	0.3245	0.07173 to 1.3467	2.1855	**0.0293**

IIEF-5, International Index of Erectile Function 5. Bold denotes statistical significance.

## Data Availability

The data presented in this study are available on request from the corresponding author.

## Abbreviations

The following abbreviations are used in this manuscript:

BMI	Body mass index
CAD	Coronary artery disease
CKD	Chronic kidney disease
ED	Erectile dysfunction
ESC	European Society of Cardiology
FSH	Follicle-stimulating hormone
GnRH	Gonadotropin-releasing hormone
IIEF-5	International Index of Erectile Function 5
IL-6	Interleukin-6
LH	Luteinizing hormone
SHBG	Sex hormone–binding globulin
TNF-α	Tumor necrosis factor-α
